# Hereditary leiomyomatosis and renal cell carcinoma: a case series and literature review

**DOI:** 10.1186/s13023-020-01653-9

**Published:** 2021-01-18

**Authors:** Zahraa Chayed, Lone Krøldrup Kristensen, Lilian Bomme Ousager, Karina Rønlund, Anette Bygum

**Affiliations:** 1 Department of Clinical Research, University of Southern Denmark, Odense, Denmark; 2 Department of Clinical Genetics, Odense University Hospital, Odense, Denmark; 3 Department of Clinical Genetics, Vejle Hospital, Vejle, Denmark; 4 Department of Dermatology and Allergy Centre, Odense University Hospital, Odense, Denmark

**Keywords:** Hereditary leiomyomatosis and renal cell carcinoma, HLRCC, Surveillance program

## Abstract

**Background:**

Hereditary leiomyomatosis and renal cell carcinoma (HLRCC) is a rare genodermatosis characterized by cutaneous leiomyoma (CLM), uterine leiomyoma (ULM) and renal cell carcinoma (RCC). Five HLRCC patients are presented with a compiled database of published HLRCC cases to increase understanding of HLRCC. Furthermore, a surveillance program is suggested. Our review is based on a PubMed search which retrieved case reports and cohort studies published before November 2019. The search yielded 97 original papers with a total of 672 HLRCC patients.

**Results:**

CLMs were present in 474 patients (71.5%), developed at the mean age of 28 years. Five patients had cutaneous leiomyosarcomas. ULMs were present in 356 women (83%), while two had uterine leiomyosarcoma. ULMs were diagnosed at a mean age of 32 years, with the youngest diagnosed at age 17 years. The most common surgical treatment for ULMs was hysterectomy, performed at a mean age of 35 years, with the youngest patient being 19 years old. RCCs were present in 189 patients (34.9%), of which half had metastatic disease. The mean age of diagnosis was 36 years with the youngest patient diagnosed with RCC at the age of 11 years.

**Conclusion:**

We suggest a surveillance program for HLRCC including a dermatological examination once every 2 years, annual magnetic resonance imaging starting at the age of 10 years to monitor for early RCCs, annual gynecological examinations from the age of 15 years and counseling regarding risk of hysterectomy and family planning at the age of 18 years. CLMs are often the earliest manifestation of HLRCC, which is why recognizing these lesions, performing a biopsy, and making a prompt referral to genetic counseling is important in order to diagnose HLRCC early.

## Background

Hereditary leiomyomatosis and renal cell carcinoma (HLRCC) is a syndrome with an autosomal dominant inheritance pattern, characterized by the development of cutaneous leiomyoma (CLM), uterine leiomyoma (ULM) and renal cell cancer (RCC) [1]. HLRCC has been reported in more than 300 families worldwide although it may be underdiagnosed [2, 3]. While CLMs are often painful, the actual morbidity of the syndrome is connected to the RCCs, which tend to be aggressive and metastasize early. ULMs in HLRCC often require hysterectomies [3, 4]. Understanding the patient group and diagnosing the disease early is therefore very important in order to adequately manage the symptoms and enroll patients in a surveillance program.

HLRCC is caused by a germline mutation in the fumarate hydratase (*FH*) gene. A pathogenic variant in *FH* can be detected in 71–100% of families with features suggestive of HLRCC [4, 5]. The *FH* gene is located on chromosome 1q43 and encodes an enzyme that catalyzes the conversion of fumarate to malate in the Krebs cycle [4–6]. Mutations in *FH* cause an accumulation of fumarate, which leads to the activation of hypoxia inducible factor 1 (HIF1). HIF1 induces expression of multiple genes involved in cell survival and proliferation, thus leading to an inappropriate activation of oncogenic hypoxia pathways [4, 7]. Biallelic mutations in FH lead to the development of fumarate hydratase deficiency, which is a rare condition characterized by neurologic dysfunction and a significantly shortened lifespan [1].

CLMs are often the first manifestation of the syndrome. They are developed at a mean age of 24 years and present as skin-colored to hyperpigmented erythematous papules or nodules, either solitary or multiple, appearing in various patterns and distributions [3–5]. CLMs are typically described in 3 different types: (1) piloleiomyomas (most common), which originate from arrector pili muscles around the hair follicles, (2) angioleiomyomas, which originate from the smooth muscles of blood vessels and (3) genital leiomyomas, which originate from the tunica dartos of the genital skin and mammary muscles of the nipples. Piloleiomyomas and angioleiomyomas tend to be painful [3, 5]. The pain can occur spontaneously or can also be induced by the application of touch or pressure, temperature changes or emotional stress. The transformation of leiomyomas to leiomyosarcomas is rare.

ULMs are benign tumors, originating from the smooth muscle of the uterus. HLRCC associated ULMs are diagnosed on an average age of 10 years earlier than sporadic fibroids, are larger and usually appear in greater numbers. ULMs often give rise to irregular menstrual cycle, menorrhagia, pain and complications related to fertility, pregnancy and birth [3–5]. Due to the severity of symptoms most patients undergo myomectomies or hysterectomies, often before the age of 30 years [4].

RCCs are tumors originating from the renal tubule epithelium. RCCs have a lower penetrance compared to the other two manifestations, as approximately 18–20% of FH mutation carriers have been diagnosed with RCC [4, 7]. The most common symptoms include hematuria, pain in the flank or lower back and a palpable mass. These tumors are more aggressive than sporadic ones and 70% of patients will die within 5 years due to disseminated disease [3–5]. RCCs tend to metastasize early, when the tumor is no larger than 1 cm. The main differential diagnoses to hereditary RCCs are Birt–Hogg–Dube syndrome (BHDS), Von Hippel–Lindau syndrome and hereditary papillary renal cancer (HPRC). BHDS is characterized by several manifestations other than RCCs, such as lung cysts and various skin lesions. VHLS is characterized by renal cysts, central nervous system hemangioblastoma and pancreatic tumors. Lastly, HPRC’s only manifestation is RCCs [5]. Another syndrome which can present with RCCs is BAP1 tumor predisposition syndrome, which is also associated with atypical Spitz tumors, uveal and cutaneous melanomas, basal cell carcinoma and malignant mesothelioma [8].

Although rare, pheochromocytomas have also been reported in relation to *FH* mutations. Patients with HLRCC are therefore likely to have an increased risk of developing pheochromocytoma [9–11].

Possible genotype–phenotype associations have been searched for but have not yet been clearly established [12, 13]. Also, a large intrafamilial variation in disease manifestations is seen, arguing against a clear genotype–phenotype correlation [14, 15].

The diagnostic criteria for HLRCC proposed by Schmidt et al. include one major criterion and three minor criteria [16]. The major criterion, which points to a high likelihood of HLRCC, is presence of multiple CLMs with at least one histologically confirmed leiomyoma. The minor criteria, which raise the suspicion of HLRCC, include: (1) solitary CLMs and a family history of HLRCC, (2) early onset type 2 papillary tumors of the kidney or (3) multiple symptomatic uterine fibroids of early onset (< 40 years old). A definitive diagnosis can be made when a pathogenic variant in *FH* is identified [16].

As patients with HLRCC have a tendency to develop early onset RCCs and symptomatic, early onset ULMs, surveillance is important in order to detect RCCs and ULMs as early as possible. Furthermore, a surveillance program should also monitor CLMs for transformation to leiomyosarcoma. Various HLRCC surveillance programs have been proposed so far, although a definitive surveillance program is yet to be established [17].

This paper presents five HLRCC patients and characterizes a series of published HLRCC cases in order to get a better understanding of this syndrome. Furthermore, a surveillance program is proposed. The following parameters will be explored: presence and characteristics of RCCs, CLMs and ULMs, age at onset or diagnosis, family history, *FH* variant status and diagnostic work-up.

## Materials and methods

This study is a review of published HLRCC case reports and cohorts. The data used in this study consists of papers collected through a literature search using PubMed. The search was conducted between 14/11/2019 and 28/11/2019 using the following keywords: HLRCC, hereditary leiomyomatosis and renal cell cancer, hereditary leiomyomatosis and renal cell carcinoma, Reed's syndrome, leiomyomatosis cutis et uteri and MCUL (multiple cutaneous leiomyomas with uterine leiomyomas). Papers included were HLRCC case reports or cohorts that described the presentation of CLMs, ULMs and RCCs. Papers were excluded if they did not contain the relevant information describing these characteristics, and if they were not in English. Some additional papers were found by searching through the reference list of other papers. In total, we identified 97 papers of which 82 were case reports and 15 were papers describing cohorts or families, with a total of 672 HLRCC cases. Data extraction was conducted by the first author, who looked for the following categories of data: gender, age of cases (at onset of disease or at diagnosis), presence and description of CLMs (age of onset, symptoms, number, location), ULMs (age at diagnosis, treatment, age at treatment) and RCCs (age at diagnosis, metastases, treatment), family history, presence of FH mutation and how the diagnosis was made (by determining what resulted in the referral: RCC symptoms, CLM symptoms, ULM symptoms or other) (Additional files 1 and 2). The data was analyzed, and mean age of onset/diagnosis were calculated by combining the data from individual case reports and the existing means from cross-sectional studies, using StataCorp 16.

## HLRCC cases

**Fig. 1:**
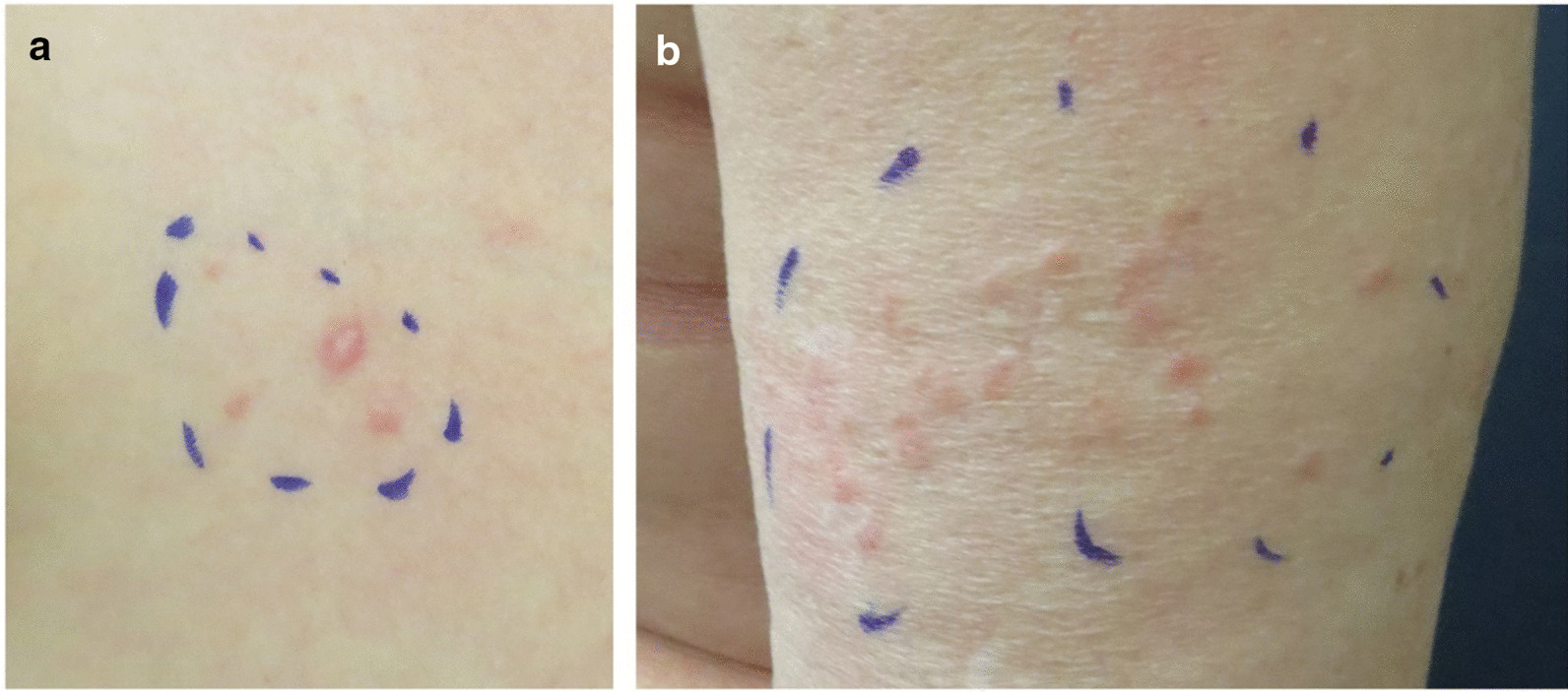
Typical appearance of cutaneous leiomyomas. **a** A single prominent red nodule, surrounded by two smaller flat lesions located at the right breast of patient 3. **b** A cluster of skin colored to reddish CLMs located on the upper arm in the mother of patient 3

See Table 1 and Fig. 1.

**Table 1:** HLRCC case presentations

	Age, gender	Family number	Family history	*Phenotype*			*Genotype*
				CLMs	ULMs	RCC	Pathogenic variant
Patient 1	55, F	I	Daughter with HLRCC (patient 2)	Multiple (> 5 CLMs)	Yes (hysterectomy at age 40)	No	c.595G > C, p.Ala199Pro
Patient 2	31, F	I	Mother with HLRCC (patient 1)	Multiple (> 5 CLMs)	No	No	c.595G > C, p.Ala199Pro
Patient 3	36, F	II	Mother with CLMs and hysterectomy due to ULMs. Maternal uncle with RCC, onset at age 65. Several family members on the maternal side with HLRCC	Multiple (> 5 CLMs)	Yes (hysterectomy at age 36)	No	c.450 T > A, p.Asn150Lys
Patient 4	41, M	III	Father with CLMs. Paternal grandmother underwent hysterectomy at the age of 28 years. Sister with HLRCC (patient 5)	Multiple (> 5 CLMs)	-	No	c.1058_1108 + 19del, p.?
Patient 5	43, F	III	Brother with HLRCC (patient 4)	Multiple (> 5 CLMs)	Yes (hysterectomy at age 28)	No	c.1058_1108 + 19del, p.?

## Results

Review of the literature resulted in inclusion of a total of 672 patients with HLRCC in our database, 239 men (35.6%) and 433 women (64.4%).

### Cutaneous leiomyoma

**Fig. 2:**
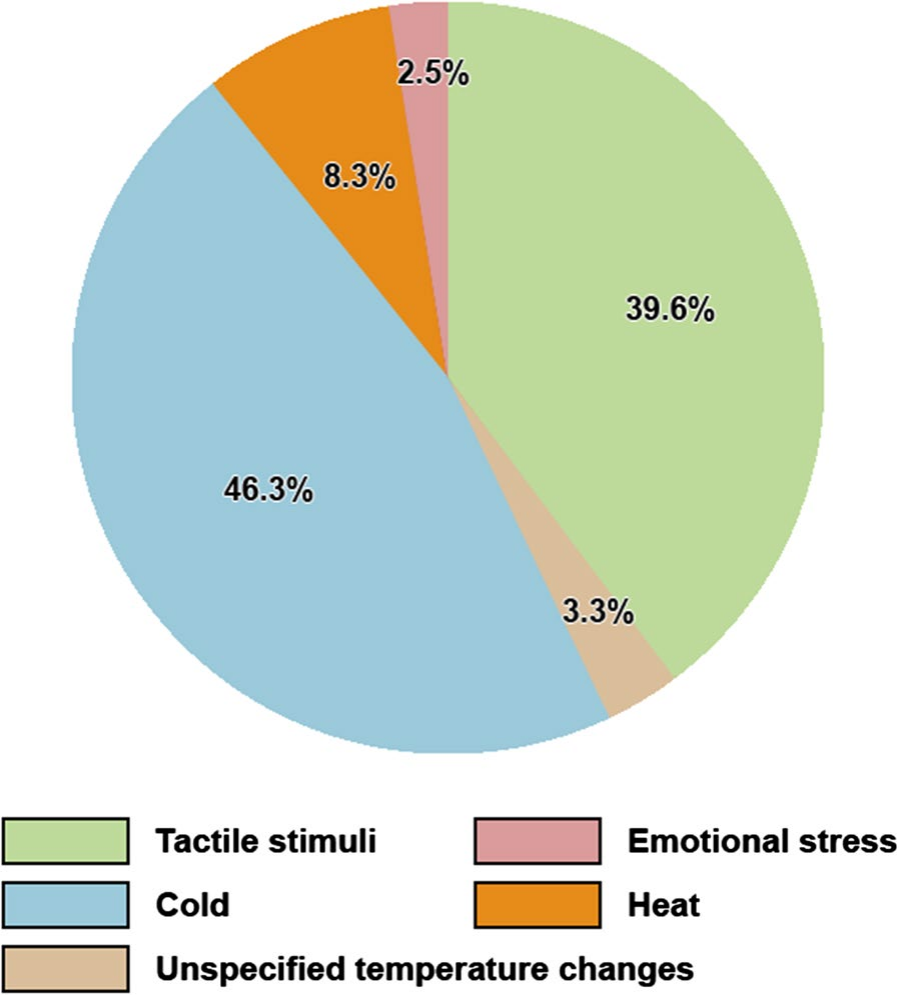
Distribution of pain triggering stimuli

**Fig. 3:**
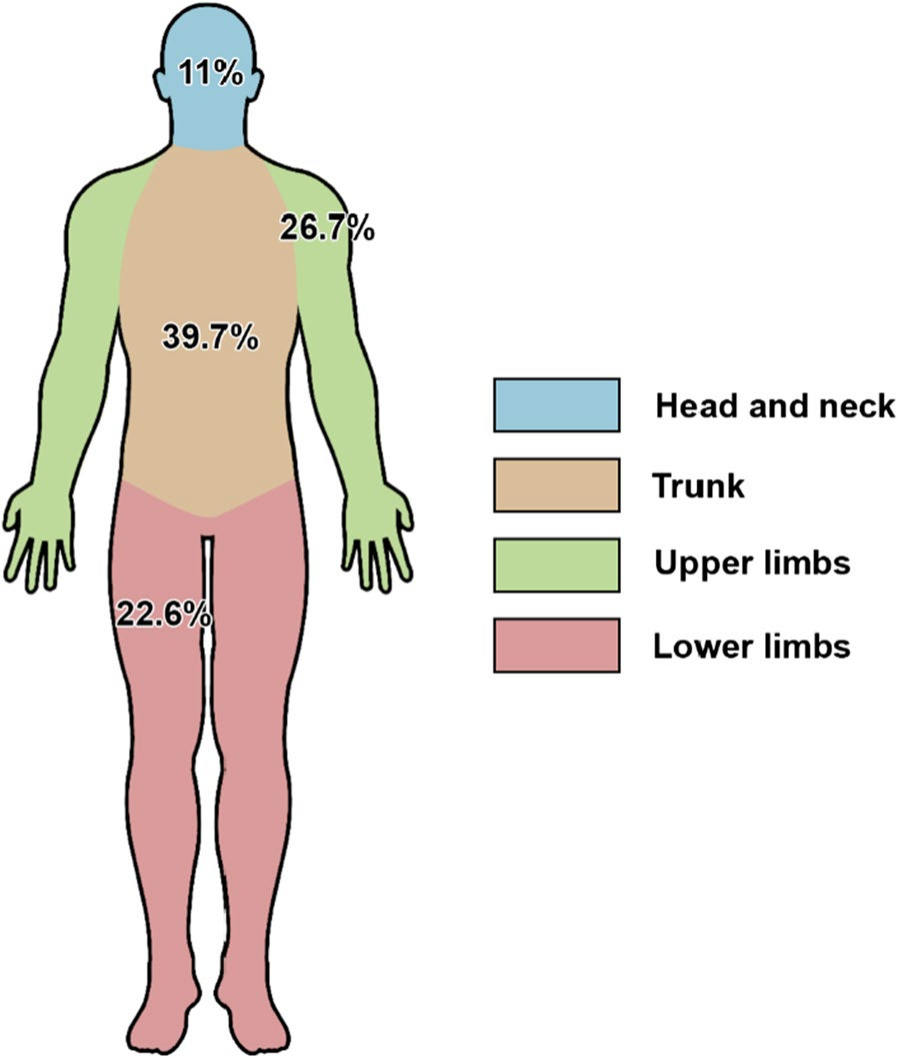
Distribution of CLMs

Table 2 provides an overview of CLMs. CLM status was reported for 663 patients (98.7%), of which 474 (71.5%) had CLMs. The mean age of onset was reported in 120 patients (25.3%) and calculated to be 28.4 years ± 11.08 standard deviation (SD) (range 11–79 years). The number of lesions was reported in 97 patients (20.5%), of which the majority had multiple (more than 5) lesions. CLM symptoms were described in 171 patients (36.1%): 31 (18.1%) were asymptomatic, 124 (72.5%) had pain, 14 (8.2%) had pruritus and two patients (1.2%) had CLMs that were both painful and pruritic. Many patients were found to have more than one stimulus that triggered pain. The distribution of these stimuli can be seen in Fig. 2. Pain in response to cold was the most common type of painful stimuli, closely followed by pain in response to tactile stimuli (touch, pressure). The location of CLMs varied widely and it was common to have lesions covering more than one location. Figure 3 shows the distribution of CLMs across various anatomical regions. A total of five patients developed cutaneous leiomyosarcomas.

**Table 2:** Overview of CLMs

Characteristic	Reported value (%)
CLM reported (n = 672)	
Yes	474 (70.5%)
No	189 (28.1%)
NA^1^	9 (1.4%)
CLM mean age at onset, mean ± SD (range)	28.4 years ± 11.08 (11–79 years) (n = 120)
CLM number	
Few (< 5)	6/474 (1.3%)
Multiple (≥ 5)	91/474 (19.2%)
NA	377/474 (79.5%)
CLM symptoms	
Asymptomatic	31/474 (6.5%)
Painful	124/474 (26.2%)
Pruritic	14/474 (3%)
Painful and pruritic	2/474 (0.4%)
NA	303/474 (63.9%)
CLM painful stimuli^2^	
Pain in response to tactile stimuli (touch, pressure)	48/121 (39.7%)
Pain in response to unspecified temperature changes	4/121 (3.3%)
Pain in response to cold	56/121 (46.3%)
Pain in response to heat	10/121 (8.2%)
Pain in response to emotional stress	3/121 (2.5%)
CLM distribution^2^	
Head and neck	33/300 (11%)
Trunk	119/300 (39.7%)
Upper limbs (including shoulders)	80/300 (26.7%)
Lower limbs (including buttocks and genitals)	68/300 (22.6%)

^1^Not available (NA) data due to lack of mention in the case reports and cohort studies

^2^CLM painful stimuli and CLM distribution were not known for all patients. Furthermore, most cases had more than one pain triggering stimuli and more than one location which was affected by CLMs.

The majority of CLMs can be found on the trunk, followed by the upper extremities, the lower extremities and lastly the head and neck area.

## Uterine leiomyoma

Table 3 provides an overview of ULMs. ULM status was known in 429 women (99.1%), of which 356 (83%) had ULMs. The age at diagnosis was known for 67 patients (18.8%) and the mean was calculated to be 32.06 years ± 6.6 SD (range 17–48 years). The choice of surgical treatment for ULM was known for 232 women (65.2%): 179 (77.2%) received a hysterectomy, 40 (17.2%) received a myomectomy and 13 (5.6%) had an unspecified surgery. Hysterectomies, the most common treatment for ULMs, were performed at a mean age of 34.7 years ± 6.2 SD (range 19–50 years). Two women developed uterine leiomyosarcomas.

**Table 3:** Overview of ULMs

ULM reported (n = 672)	
Yes	356/433 (82.2%)
No	73/433 (16.9%)
NA	4/433 (0.9%)
ULM age at diagnosis mean ± SD (range)	32.06 years ± 6.6 (17–48 years) (n = 67)
ULM treatment	
Myomectomy	40/356 (11.2%)
Hysterectomy	179/356 (50.3%)
Unspecified surgery	13/356 (3.7%)
NA	124/356 (34.8%)
ULM age at hysterectomy, mean ± SD (range)	34.7 years ± 6.2 (19–50 years) (n = 60)

## Renal cell carcinoma

Table 4 provides an overview of RCCs. RCC status was known for 542 patients (80.7%) of which 189 (34.9%) had RCCs, 101 (53.4%) in a metastatic state. The mean age of diagnosis for RCC was reported in 51 patients (27%) and calculated to be 36.1 years ± 13.4 SD (range 11 –79 years).

**Table 4:** Overview of RCCs

RCC reported (n = 672)	
Yes	189 (28.1%)
No	353 (52.5%)
NA	130 (19.4%)
RCC mean age at diagnosis, mean ± SD (range)	36.1 years ± 13.4 (11–79 years) (n = 51)
RCC metastases	
Yes	101/189 (53.5%)
No	49/189 (25.9%)
NA	39/189 (20.6%)
RCC treatment	
Nephrectomy (unspecified type)	31/189 (16.4%)
Nephrectomy (radical)	25/189 (13.2%)
Nephrectomy (partial)	6/189 (3.2%)
NA	127/189 (67.2%)

## Family history, FH variants and diagnosis

The majority of the patients had a positive family history for RCCs, CLMs or ULMs. Of the 227 patients who had been asked about family history, 199 (87.8%) could confirm a positive family history. *FH* mutational testing was conducted in 333 patients and revealed a pathogenic variant in 332 (99.7%) of those. In the remaining patient, sequencing of the FH exon regions (using RNA extracted from a leiomyoma) did not reveal any genetic variants; however, this does not exclude gross deletions or the presence of a pathogenic variant in the promotor region or in the intron regions.

**Fig. 4:**
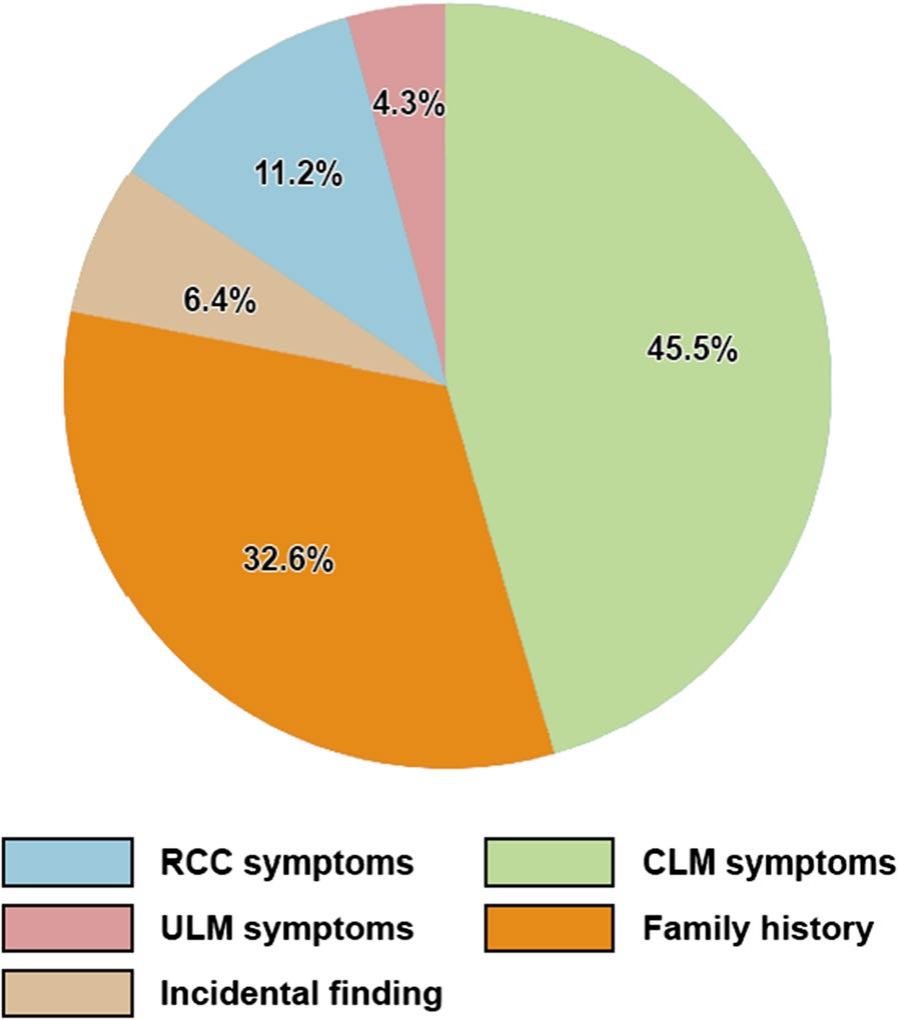
Overview of cardinal symptoms leading to a diagnosis of HLRCC

Figure 4 gives an overview of how the clinical diagnosis was made: 85 patients (45.5%) were diagnosed based on CLM symptoms, 21 (11.2%) were diagnosed due to RCC symptoms, 8 (4.3%) were diagnosed due to ULM symptoms, 61 (32.6%) were diagnosed due to a positive family history or the diagnosis of a close family member and lastly 12 (6.4%) were diagnosed due an incidental finding of HLRCC manifestations.

## Discussion

We conducted a literature search and characterized the clinical features of 672 HLRCC patients. Based on our findings we propose the surveillance program for HLRCC presented in Table 5 and discussed below.

**Table 5:** Proposed HLRCC surveillance program for confirmed *FH* mutation carriers

CLM
Dermatological examination once every 2 years, starting from onset of CLMs
Instructions to contact a dermatologist in case of changes in CLMs
ULM
Annual gynecological ultrasound examination, starting from the age of 15 years
Counseling regarding risk of hysterectomy and family planning at the age of 18 years
RCC
Annual contrast enhanced MRI of the abdomen, starting from the age of 10 years

We found that CLMs were often the earliest manifestation of HLRCC, appearing at a mean age of 28.4 years. In a study by Toro et al. CLMs appeared at a mean age of 25 years [18]. This makes these cutaneous lesions very important to recognize in order to diagnose the disease early. With CLM symptoms also being the most common symptoms, that lead to a diagnosis of HLRCC, this further highlights the importance of recognizing CLMs, performing a biopsy and making a prompt referral to genetic counseling to uncover family history and test for *FH* variants. The majority of CLMs in our study were described as painful, but this could perhaps be due to selection bias as patients with asymptomatic CLMs are less likely to seek medical intervention and thus less likely to be reported. We found five cases of cutaneous leiomyosarcomas in our study. For early detection of CLM transformation into leiomyosarcomas, Patel et al. recommends dermatological examination annually or once every 2 years in patients diagnosed with HLRCC [3]. With the low prevalence of cutaneous leiomyosarcomas in mind, we suggest that a dermatological examination once every 2 years is sufficient. Furthermore, it is important to notify patients to seek a dermatologist if they notice a change in their lesions.

We found that 83% of women in our study had ULMs, diagnosed at a mean age of 32.06 years. This indicates that ULMs are a highly penetrant manifestation of HLRCC, and is supported by other studies, which show that 81.7%-98% of women with *FH* mutations are affected with ULMs [7, 18]. Sporadic fibroids, in comparison, have been found to have an overall prevalence of 9.6% in the general population with the highest incidence in age group 45–49 years [19]. This means that HLRCC related ULMs are diagnosed approximately 13–17 years earlier than their sporadic counterparts. ULMs pose a significant risk to fertility in themselves but also due to their highly symptomatic nature which often require early hysterectomies. This study found that 77.2% of women with ULMs had a hysterectomy performed at the mean age of 34.7 years. A Portuguese study found the mean age of hysterectomies in the general population to be approximately 55.2 years, while the mean age of hysterectomies due to sporadic uterine fibroids was 48.6 years [20]. This means that hysterectomies due to HLRCC associated ULMs are performed 14–21 years earlier than in the general population. The high risk of early hysterectomies raises a serious concern for the affected women’s fertility, which is why Smit et al. argues that these patients should receive early adulthood counseling on family planning [21]. The youngest patient to undergo hysterectomy in our study was 19 years of age. Therefore, we suggest that the optimal time for consultation regarding risk of hysterectomy and family planning would be at the age of 18 years. With the age of the youngest patient diagnosed with ULMs in mind, we suggest that the surveillance program includes annual gynecological examinations starting from the age of 15 years. The recommended method of surveillance is ultrasound examination [21].

HLRCC associated RCCs have long been known to develop at early age and to have early metastatic potential. Additionally, HLRCC-associated RCCs can metastasize when the tumor is no larger than 1 cm. The mean age of diagnosis found in our study confirms the early development of RCCs. To emphasize how early these tumors develop, it is worth noting that sporadic RCCs have a mean age of diagnosis of 63–68 years [22, 23], which is approximately 27–32 years later than HLRCC associated RCCs. This highlights the necessity of an early screening program. Patel et al. recommends starting the screening program at the age of 10 years while Schmidt et al. recommends starting as early as 8 years [3, 16]. Menko et al. recommends that decisions about renal surveillance before the age of 18 years should be made on an individual basis [17]. However, we found four patients in our study who developed RCC before the age of 20, the youngest being 11 years old, while a recent review identified 7 more patients, the youngest being 10 years old, which is why we will also recommend starting the screening program at the age of 10 years [24]. The method for surveillance should be annual contrast enhanced MRI of the kidneys [2–4]. Lastly, it is worth pointing out that our review found 34.9% of the patients to have RCCs while previous studies have showed a penetrance of approximately 18–20%. This higher prevalence is likely caused by publication bias as cases with RCCs are more likely to be reported on.

To start surveillance early would also require early genetic testing. Genetic testing of children always implicates important concerns on best interest and autonomy (including loss of their own adult choice about knowing their genetic status). Decisions to test must always balance the likely benefits and risks. The suggested surveillance program aims for early detection of tumors at a more favorable prognostic level. We believe that the child and parents should be offered genetic counselling and FH variant test before entry in the surveillance program. Informed consent should be given by the parents and the child should be engaged in the decision at a developmentally appropriate level, in keeping with ethics guidance.

In this study we found that the majority of patients with HLRCC had a positive family history. This highlights the need to inquire about the family history of CLMs, ULMs (including hysterectomies), and RCCs in patients who are suspected of having HLRCC. Especially the gynecologists should be alert and ask for a personal and familial history regarding skin and renal tumors, when they treat women with early onset ULMs. A positive family history, together with cutaneous leiomyomas, is one of the minor diagnostic criteria proposed by Schmidt et al. to make the HLRCC diagnosis [16]. A diagnosis of HLRCC can definitively be made when a germline *FH* mutation is found. In this review we found that 333 patients with characteristics indicative of HLRCC were tested genetically and of these 99.7% had a pathogenic *FH* variant. Earlier data showed germline *FH* variants in ~ 90% (76–100%) of families with clinical features suggestive of HLRCC [4]. Genetic testing of the *FH* gene is strongly recommended in order to confirm the diagnosis and be able to identify family members who should also be offered genetic counseling, genetic test, and enrolled in the suggested surveillance program if they are found to be carriers of a detected *FH* variant. Based on the above information, we have outlined our recommendations for *FH* genetic testing in Table 6. In the few patients with clinical HLRCC wherein a pathogenic *FH* variant can't be detected, additional immunohistochemical studies of tumors may support the diagnosis [25].

**Table 6:** Recommendations for *FH* genetic testing

Test for FH variants should be carried out in patients with
More than one cutaneous leiomyoma
One or more cutaneous leiomyomas and uterine leiomyomas (females)
One or more leiomyoma and renal tumor
One or more leiomyoma (cutaneous or uterine) and 1st degree relatives with multiple cutaneous leiomyomas, uterine leiomyomas or renal tumors

The study is based on published case reports and case series, which are prone to selection bias, as more severely affected patients may have been reported with a focus on the organ manifestations of interest of the author group. Most were cross-sectional studies, which might skew the results towards the earlier appearing manifestations, as the disease manifestations are age related and especially RCC may develop later in life. We may also have overlooked some patient reports in the literature.

Finally, our review included 239 men and 433 women. The big difference in number might point to an underdiagnosis of HLRCC in men, although that cannot be confirmed. Only two HLRCC manifestations are seen in men, CLMs and RCCs, which could perhaps limit their diagnosis options when compared to women. Case reports for women could be found in gynecology, urology and dermatology journals which might explain the overrepresentation of women.

## Conclusion

HLRCC is a rare genodermatosis, yet important to know and identify due to its morbidity and high risk of early malignancy, which also stresses the necessity of an appropriate surveillance program.

We suggest a dermatological examination once every two years as part of the surveillance program to monitor for transformation of CLMs to leiomyosarcoma. CLMs were found to be the first manifestation of HLRCC and the most common symptom that led to the diagnosis. It is therefore important that physicians are able to recognize these lesions based on patient symptoms and clinical appearance combined with histopathology, and to immediately suspect HLRCC in order to diagnose the disease early.

Furthermore, ULMs are diagnosed approximately 13–17 years earlier than their sporadic counterparts and have a great impact on fertility, which is why we suggest annual gynecological examinations from the age of 15 years and a counseling regarding risk of hysterectomy and family planning at the age of 18 years. Finally, we suggest an annual contrast enhanced MRI of the kidneys from the age of 10 years in order to find RCCs at an early stage.

## Supplementary information

**Additional file 1.** Case database 1. This database includes data from case reports about HLRCC patients. The data includes details about gender, age, presence and description of CLMs, ULMs and RCCs etc. The results of our review are based on this database and database 2.

**Additional file 2.** Case database 2. This database includes grouped data from cohort series about HLRCC patients. The data includes details about gender, age, presence and description of CLMs, ULMs and RCCs etc.

**Additional file 3.** Supplemental bibliography. This file includes the references to the 97 original papers from which our data was collected.

## Data Availability

Apart from the five new cases presented, all data used for this paper was from publicly available sources (PubMed). A list of the original publications from which the data is collected is provided in Additional file 3.

## Abbreviations

CLM: cutaneous leiomyoma
FH: fumarate hydratase
HLRCC: hereditary leiomyoma and renal cell carcinoma
RCC: renal cell carcinoma
ULM: uterine leiomyoma

## References

[1] Alam NA, Barclay E, Rowan AJ, Tyrer JP, Calonje E, Manek S (2005). Clinical features of multiple cutaneous and uterine leiomyomatosis: an underdiagnosed tumor syndrome. Arch Dermatol.

[2] Kamihara J, Schultz KA, Rana HQ. FH Tumor Predisposition Syndrome. 2006 Jul 31 [updated 2020 Apr 2]. In: Adam MP, Ardinger HH, Pagon RA, Wallace SE, Bean LJH, Stephens K, Amemiya A, editors. GeneReviews^®^ [Internet]. Seattle (WA): University of Washington, Seattle; 1993–2020. Accessed 10 Jan 2020.

[3] Patel VM, Handler MZ, Schwartz RA, Lambert WC (2017). Hereditary leiomyomatosis and renal cell cancer syndrome: an update and review. J Am Acad Dermatol.

[4] Lehtonen HJ (2011). Hereditary leiomyomatosis and renal cell cancer: update on clinical and molecular characteristics. Fam Cancer.

[5] Hansen AW, Chayed Z, Pallesen K, Codruta Vasilescu I, Bygum A (2020). Hereditary Leiomyomatosis and Renal Cell Cancer. Acta Derm Venereol..

[6] Brown S, Brennan P, Rajan N (2017). Inherited skin tumour syndromes. Clin Med (Lond).

[7] Gardie B, Remenieras A, Kattygnarath D, Bombled J, Lefèvre S, Perrier-Trudova V (2011). Novel FH mutations in families with hereditary leiomyomatosis and renal cell cancer (HLRCC) and patients with isolated type 2 papillary renal cell carcinoma. J Med Genet.

[8] Pilarski R, Carlo M, Cebulla C, Abdel-Rahman M. BAP1 Tumor Predisposition Syndrome. 2016 Oct 13 [updated 2020 Sep 17]. In: Adam MP, Ardinger HH, Pagon RA, Wallace SE, Bean LJH, Stephens K, Amemiya A, editors. GeneReviews® [Internet]. Seattle (WA): University of Washington, Seattle; 1993–2020. Accessed 21 Oct 2020. 27748099

[9] Ben Aim L, Pigny P, Castro-Vega LJ, Buffet A, Amar L, Bertherat J (2019). Targeted next-generation sequencing detects rare genetic events in pheochromocytoma and paraganglioma. J Med Genet.

[10] Udager AM, Magers MJ, Goerke DM, Vinco ML, Siddiqui J, Cao X (2018). The utility of SDHB and FH immunohistochemistry in patients evaluated for hereditary paraganglioma-pheochromocytoma syndromes. Hum Pathol.

[11] Clark GR, Sciacovelli M, Gaude E, Walsh DM, Kirby G, Simpson MA (2014). Germline FH mutations presenting with pheochromocytoma. J Clin Endocrinol Metab.

[12] Wei MH, Toure O, Glenn GM, Pithukpakorn M, Neckers L, Stolle C (2006). Novel mutations in FH and expansion of the spectrum of phenotypes expressed in families with hereditary leiomyomatosis and renal cell cancer. J Med Genet.

[13] Bhola PT, Gilpin C, Smith A, Graham GE (2018). A retrospective review of 48 individuals, including 12 families, molecularly diagnosed with hereditary leiomyomatosis and renal cell cancer (HLRCC). Fam Cancer.

[14] Guinard E, Legendre L, Kramkimel N, Avril MF, Chassaing N, Cabaret O (2016). Complete penetrance and absence of intrafamilial variability in a large family with hereditary leiomyomatosis and renal cell carcinoma. Dermatology.

[15] Vocke CD, Ricketts CJ, Merino MJ, Srinivasan R, Metwalli AR, Middelton LA (2017). Comprehensive genomic and phenotypic characterization of germline FH deletion in hereditary leiomyomatosis and renal cell carcinoma. Genes Chromosomes Cancer.

[16] Schmidt LS, Linehan WM (2014). Hereditary leiomyomatosis and renal cell carcinoma. Int J Nephrol Renovasc Dis.

[17] Menko FH, Maher ER, Schmidt LS, Middelton LA, Aittomäki K, Tomlinson I (2014). Hereditary leiomyomatosis and renal cell cancer (HLRCC): renal cancer risk, surveillance and treatment. Fam Cancer.

[18] Toro JR, Nickerson ML, Wei MH, Warren MB, Glenn GM, Turner ML (2003). Mutations in the fumarate hydratase gene cause hereditary leiomyomatosis and renal cell cancer in families in North America. Am J Hum Genet.

[19] Yu O, Scholes D, Schulze-Rath R, Grafton J, Hansen K, Reed SD (2018). A US population-based study of uterine fibroid diagnosis incidence, trends, and prevalence: 2005 through 2014. Am J Obstet Gynecol.

[20] Gante I, Medeiros-Borges C, Águas F (2017). Hysterectomies in Portugal (2000–2014): What has changed?. Eur J Obstet Gynecol Reprod Biol.

[21] Smit DL, Mensenkamp AR, Badeloe S, Breuning MH, Simon ME, van Spaendonck KY (2011). Hereditary leiomyomatosis and renal cell cancer in families referred for fumarate hydratase germline mutation analysis. Clin Genet.

[22] Karakiewicz PI, Jeldres C, Suardi N, Hutterer GC, Perrotte P, Capitanio U (2008). Age at diagnosis is a determinant factor of renal cell carcinoma-specific survival in patients treated with nephrectomy. Can Urol Assoc J.

[23] Doehn C, Grünwald V, Steiner T, Follmann M, Rexer H, Krege S (2016). The diagnosis, treatment, and follow-up of renal cell carcinoma. Dtsch Arztebl Int.

[24] Hol JA, Jongmans MCJ, Littooij AS, de Krijger RR, Kuiper RP, van Harssel JJT (2020). Renal cell carcinoma in young FH mutation carriers: case series and review of the literature. Fam Cancer.

[25] Bardella C, El-Bahrawy M, Frizzell N, Adam J, Ternette N, Hatipoglu E (2011). Aberrant succination of proteins in fumarate hydratase-deficient mice and HLRCC patients is a robust biomarker of mutation status. J Pathol.

