# Supplementary material for: Hereditary leiomyomatosis and renal cell carcinoma: a case series and literature review
Source: Orphanet J Rare Dis. 2021 Jan 18;16:34. doi: 10.1186/s13023-020-01653-9 (PMC7814596; doi:10.1186/s13023-020-01653-9)
Supplement: Supplementary file 3 — Additional file 3. Supplemental bibliography. This file includes the references to the 97 original papers from which our data was collected. [file 13023_2020_1653_MOESM3_ESM.docx]

## Additional file 3: Supplemental bibliography

1. Adamane S, Desai S, Menon S. Hereditary leiomyomatosis and renal cell cancer syndrome associated renal cell carcinoma. Indian J Pathol Microbiol. 2017;60:108-110.
2. Adams A, Sharpe KK, Peters P, Freeman M. Hereditary leiomyomatosis and renal cell cancer (HLRCC): cutaneous and renal manifestations requiring a multidisciplinary team approach. BMJ Case Rep. 2017;2017:bcr2016215115.
3. Akita F, Kambe N, Nakano M, Satoh T, Iwasawa M, Togawa Y, et al. Novel R218X mutation in the fumarate hydratase gene in a patient with Reed's syndrome. J Dermatol. 2013;40:58-9.
4. Alam NA, Barclay E, Rowan AJ, Tyrer JP, Calonje E, Manek S, et al. Clinical features of multiple cutaneous and uterine leiomyomatosis: an underdiagnosed tumor syndrome. Arch Dermatol. 2005;141:199-206.
5. Alam M, Rabinowitz AD, Engler DE. Gabapentin treatment of multiple piloleiomyoma-related pain. J Am Acad Dermatol. 2002;46:S27‐9.
6. Almeida FT, Santos RP, Carvalho SD, Brito MC. Reed's Syndrome. Indian J Dermatol. 2018;63:261‐3.
7. Alrashdi I, Levine S, Paterson J, Saxena R, Patel SR, Depani S, et al. Hereditary leiomyomatosis and renal cell carcinoma: very early diagnosis of renal cancer in a paediatric patient. Fam Cancer. 2010;9:239-43.
8. Arenas Valencia C, Rodríguez López ML, Cardona Barreto AY, Garavito Rodríguez E, Arteaga Díaz CE. Hereditary leiomyomatosis and renal cell cancer syndrome: identification and clinical characterization of a novel mutation in the FH gene in a Colombian family. Fam Cancer. 2017;16:117-22.
9. Arora R, Eble JN, Pierce HH, Crispen PL, DeSimone CP, Lee EY, et al. Bilateral ovarian steroid cell tumours and massive macronodular adrenocortical disease in a patient with hereditary leiomyomatosis and renal cell cancer syndrome. Pathology. 2012;44:360-3.
10. Basendwh MA, Fatani M, Baltow B. Reed's Syndrome: A Case of Multiple Cutaneous Leiomyomas Treated with Liquid Nitrogen Cryotherapy. Case Rep Dermatol. 2016;8:65-70.
11. Bava EP, Sharma A, Chumber S, Anand RK. Gastrointestinal Stromal Tumour in a Patient with Multiple Cutaneous and Uterine Leiomyomatosis- Implications and Anaesthetic Management. Indian J Surg Oncol. 2015;6:106-9.
12. Behnes CL, Schlegel C, Shoukier M, Magiera I, Henschke F, Schwarz A, et al. Hereditary papillary renal cell carcinoma primarily diagnosed in a cervical lymph node: a case report of a 30-year-old woman with multiple metastases. BMC Urol. 2013;13:3.
13. Bevans SL, Mayo TT, Pavlidakey PG, Cannon AD, Korf BR, Mercado PJ. Unusual presentation of hereditary leiomyomatosis mimicking neurofibromatosis. JAAD Case Rep. 2018;4:440-1.
14. Bhola PT, Gilpin C, Smith A, Graham GE. A retrospective review of 48 individuals, including 12 families, molecularly diagnosed with hereditary leiomyomatosis and renal cell cancer (HLRCC). Fam Cancer. 2018;17:615-20.
15. Çaliskan E, Bodur S, Ulubay M, Özmen I, Çiçek AF, Deveci G, et al. Hereditary leiomyomatosis and renal cell carcinoma syndrome: a case report and implications of early onset. An Bras Dermatol. 2017;92:88-91.
16. Cantisani C, Miraglia E, Giustini S, Calvieri S. Multiple painful papulo-nodular lesions: clinical pitfall. Clin Ter. 2014;165:e423-5.
17. Cassetty CT. Familial leiomyomatosis cutis et uteri. Dermatol Online J. 2004;10:5.
18. Chan I, Wong T, Martinez-Mir A, Christiano AM, McGrath JA. Familial multiple cutaneous and uterine leiomyomas associated with papillary renal cell cancer. Clin Exp Dermatol. 2005;30:75-8.
19. Chinniah N, Lowe P. Hereditary leiomyomatosis and renal cell carcinoma syndrome. Med J Aust. 2015;203:78-9.
20. Collgros H, Iglesias-Sancho M, Tribó-Boixareu MJ, Creus-Vila L, Umbert-Millet P, Salleras-Redonnet M. Multiple cutaneous and uterine leiomyomatosis or reed syndrome: a retrospective study of 13 cases. Actas Dermosifiliogr. 2015;106:117-25.
21. Costella TM, Romiti N, Almeida JR, Dinato SL, Sementilli A, Ciofi TP. Do you know this syndrome? An Bras Dermatol. 2011;86:815.
22. da Cunha IW, da Costa WH, Morini MA, Bezerra SM, Carraro DM, Torrezan GT, et al. Expanding morphological and clinical aspects of hereditary leiomyomatosis and renal cell carcinoma (HLRCC): a case report in a patient with unusual morphology and clinical presentation. Virchows Arch. 2018;473:775-9.
23. Da Silva DM, Roth RR, Simpson CL. Teledermatology leading to an important diagnosis in an underserved clinic. Dermatol Online J. 2018;24:13030/qt3nf839r6.
24. Deshmukh P, Sharma YK, Chaudhari ND, Dash K, Mulay P. Familial myomatosis cutis et uteri, segmental type 2. Indian Dermatol Online J. 2013;4:309-10.
25. Diluvio L, Torti C, Terrinoni A, Candi E, Piancatelli R, Piccione E, et al. Dermoscopy as an adjuvant tool for detecting skin leiomyomas in patient with uterine fibroids and cerebral cavernomas. BMC Dermatol. 2014;14:7.
26. Duong BT, Savarirayan R, Winship I. Incidental diagnosis of HLRCC following investigation for Asperger Syndrome: actionable and actioned. Fam Cancer. 2016;15:25-9.
27. Elbuluk N, Bichakjian CK, Lowe L. What is your diagnosis? Multiple cutaneous and uterine leiomyomatosis (Reed Syndrome). Cutis. 2011;87:65, 76-7.
28. Emer JJ, Solomon S, Mercer SE. Reed's Syndrome: A Case of Multiple Cutaneous and Uterine Leiomyomas. J Clin Aesthet Dermatol. 2011;4:37-42.
29. Engelke H, Christophers E. Leiomyomatosis cutis et uteri. Acta Derm Venereol Suppl (Stockh). 1979;59:51‐4.
30. Fondriest SA, Gowdy JM, Goyal M, Sheridan KC, Wasdahl DA. Concurrent renal-cell carcinoma and cutaneous leiomyomas: A case of HLRCC. Radiol Case Rep. 2015;10:962.
31. Frey MK, Worley MJ Jr, Heyman KP, Caputo TA. A case report of hereditary leiomyomatosis and renal cell cancer. Am J Obstet Gynecol. 2010;202:e8‐9.
32. Garg K, Tickoo SK, Soslow RA, Reuter VE. Morphologic features of uterine leiomyomas associated with hereditary leiomyomatosis and renal cell carcinoma syndrome: a case report. Am J Surg Pathol. 2011;35:1235-7.
33. Grubb RL 3rd, Franks ME, Toro J, Middelton L, Choyke L, Fowler S, et al. Hereditary leiomyomatosis and renal cell cancer: a syndrome associated with an aggressive form of inherited renal cancer. J Urol. 2007;177:2074-80.
34. Guinard E, Legendre L, Kramkimel N, Avril MF, Chassaing N, Cabaret O, et al. Complete Penetrance and Absence of Intrafamilial Variability in a Large Family with Hereditary Leiomyomatosis and Renal Cell Carcinoma. Dermatology. 2016;232:293-7.
35. Gunnala V, Pereira N, Irani M, Lilienthal D, Pirog EC, Soslow R, et al. Novel Fumarate Hydratase Mutation in Siblings With Early Onset Uterine Leiomyomas and Hereditary Leiomyomatosis and Renal Cell Cancer Syndrome. Int J Gynecol Pathol. 2018;37:256-61.
36. Gupta G, Sudan R, Mushtaq S. Multiple Cutaneous and Uterine Leiomyomatosis with Renal Involvement: Report of a Rare Association. Indian J Dermatol. 2018;63:73-5.
37. Harris M, Wallace J, Winship I, Hale L, Gardner M. Hereditary renal cell carcinoma: the clue can be in the skin. Intern Med J. 2009;39:e12-3.
38. Hayedeh G, Fatemeh M, Ahmadreza R, Masoud A, Ahmad S. Hereditary leiomyomatosis and renal cell carcinoma syndrome: a case report. Dermatol Online J. 2008;14:16.
39. Henley ND, Tokarz VA. Multiple cutaneous and uterine leiomyomatosis in a 36-year-old female, and discussion of hereditary leiomyomatosis and renal cell carcinoma. Int J Dermatol. 2012;51:1213-6.
40. Hsu T, Cornelius LA, Rosman IS, Nemer KM. Treatment of cutaneous leiomyomas with 5% lidocaine patches in a patient with hereditary leiomyomatosis and renal cell cancer (Reed syndrome). JAAD Case Rep. 2017;3:407-9.
41. Hüller C, Grunow N, Nadler T, Bär M. Cutaneous and uterine leiomyomatosis and ovarian cystadenoma associated with deficiency of fumarate hydratase. Dermatol Pract Concept. 2011;1:25-7.
42. Kakar R, Davis JC, Crowe DR. Multiple linear leiomyomas of the forehead as the presenting sign of Reed syndrome. Int J Dermatol. 2014;53:316-8.
43. Kim G. Multiple cutaneous and uterine leiomyomatosis (Reed's syndrome). Dermatol Online J. 2005;11:21.
44. Kontochristopoulos G, Kouris A, Balamoti E, Vavouli C, Markantoni V, Christofidou E, et al. A Case of Reed's Syndrome: An Underdiagnosed Tumor Disorder. Case Rep Dermatol. 2014;6:189-93.
45. Kopp RP, Stratton KL, Glogowski E, Schrader KA, Rau-Murthy R, Russo P, et al. Utility of prospective pathologic evaluation to inform clinical genetic testing for hereditary leiomyomatosis and renal cell carcinoma. Cancer. 2017;123:2452-8.
46. Launonen V, Vierimaa O, Kiuru M, Isola J, Roth S, Pukkala E, et al. Inherited susceptibility to uterine leiomyomas and renal cell cancer. Proc Natl Acad Sci U S A. 2001;98:3387-92.
47. Lee JJ, Nambudiri VE, Henneberry J, Larson AR, Wanner M. Post-transplant diagnosis of hereditary leiomyomatosis and renal cell carcinoma syndrome in a kidney donor. Clin Kidney J. 2014;7:615-6.
48. Lehtonen HJ, Blanco I, Piulats JM, Herva R, Launonen V, Aaltonen LA. Conventional renal cancer in a patient with fumarate hydratase mutation. Hum Pathol. 2007;38:793-6.
49. Lencastre A, Cabete J, Gonçalves R, João A, Fidalgo A. Cutaneous leiomyomatosis in a mother and daughter. An Bras Dermatol. 2013;88:124-7.
50. Mandal RK, Koley S, Banerjee S, Kabiraj SP, Ghosh SK, Kumar P. Familial leiomyomatosis cutis affecting nine family members in two successive generations including four cases of Reed's syndrome. Indian J Dermatol Venereol Leprol. 2013;79:83-7.
51. Mann ML, Ezzati M, Tarnawa ED, Carr BR. Fumarate Hydratase Mutation in a Young Woman With Uterine Leiomyomas and a Family History of Renal Cell Cancer. Obstet Gynecol. 2015;126:90-2.
52. Matsuda T, Kambe N, Ly NTM, Ueda-Hayakawa I, Yamazaki F, Ohe C, et al. Hereditary leiomyomatosis and renal cell cancer syndrome in which skin biopsy enabled diagnosis. J Dermatol. 2019;46:e285-7.
53. McKelvey KD Jr, Siraj S, Kelsay J, Batres F. Male infertility associated with hereditary leiomyomatosis and renal cell carcinoma. Fertil Steril. 2010;93:2075.e1-2.
54. Mehrtens S, Veitch D, Kulakov E, Perrett CM. A Case of Hereditary Leiomyomatosis and Renal Cell Carcinoma. Case Rep Dermatol Med. 2016;2016:3793986.
55. Merino MJ, Torres-Cabala C, Pinto P, Linehan WM. The morphologic spectrum of kidney tumors in hereditary leiomyomatosis and renal cell carcinoma (HLRCC) syndrome. Am J Surg Pathol. 2007;31:1578-85.
56. Michelon MA, Layton CJ, Jessup CJ, Lizzul PF. Cutaneous clues to renal cell carcinoma: hereditary leiomyomatosis and renal cell carcinoma. J Drugs Dermatol. 2013;12:578-9.
57. Millán-Cayetano JF, Segura-Palacios JM, de-Troya-Martín M. Painful skin nodules. Actas Dermosifiliogr. 2015;106:423-4.
58. Mitchum MD, Adams EG, Holcomb KZ. JAAD Grand Rounds quiz. A 46-year-old man with agminated papules on the buttock. Reed syndrome. J Am Acad Dermatol. 2012;66:337-9.
59. Muller M, Ferlicot S, Guillaud-Bataille M, Le Teuff G, Genestie C, Deveaux S, et al. Reassessing the clinical spectrum associated with hereditary leiomyomatosis and renal cell carcinoma syndrome in French FH mutation carriers. Clin Genet. 2017;92:606-15.
60. Nagarajan P, Kenney B, Drost P, Galan A. An unusual case of sporadic hereditary leiomyomatosis and renal cell carcinoma syndrome. Cutis. 2015;95:E7-9.
61. Natália F, Tiago O, Pedro O, Sandro G. Hereditary leiomyomatosis and renal cell carcinoma: Case report and review of the literature. Urol Ann. 2018;10:108-10.
62. Noguchi G, Furuya M, Okubo Y, Nagashima Y, Kato I, Matsumoto K, et al. Hereditary leiomyomatosis and renal cell cancer without cutaneous manifestations in two Japanese siblings. Int J Urol. 2018;25:832-5.
63. Orseth ML, Redick D, Pinczewski J, Wilson BB, Kuppalli SS. Something to Reed about: fibroids, cutaneous leiomyomas, and renal cell carcinoma. Am J Obstet Gynecol. 2014;210:584.e1-2.
64. Pan X, Zhang M, Yao J, Zeng H, Nie L, Gong J, et al. Fumaratehydratase-deficient renal cell carcinoma: a clinicopathological and molecular study of 13 cases. J Clin Pathol. 2019;72:748-54.
65. Park I, Shim YS, Go H, Hong BS, Lee JL. Long-term response of metastatic hereditary leiomyomatosis and renal cell carcinoma syndrome associated renal cell carcinoma to bevacizumab plus erlotinib after temsirolimus and axitinib treatment failures. BMC Urol. 2019;19:51.
66. Perkins J, Scarbrough C, Sammons D, Magro C. Reed syndrome: an atypical presentation of a rare disease. Dermatol Online J. 2014;21:13030/qt5k35r5pn.
67. Popadić M, Brasanac D, Milinković M, Milčić D. Dermatoscopy of multiple piloleiomyomas with disseminated and segmental distribution. Indian J Dermatol Venereol Leprol. 2018;84:726-9.
68. Rai VM, Balachandran C, Kudva R. Multiple painful nodules. Indian J Dermatol Venereol Leprol. 2005;71:449-51.
69. Raymond VM, Herron CM, Giordano TJ, Gruber SB. Familial renal cancer as an indicator of hereditary leiomyomatosis and renal cell cancer syndrome. Fam Cancer. 2012;11:115-21.
70. Refae MA, Wong N, Patenaude F, Bégin LR, Foulkes WD. Hereditary leiomyomatosis and renal cell cancer: an unusual and aggressive form of hereditary renal carcinoma. Nat Clin Pract Oncol. 2007;4:256-61.
71. Rieder E, Shvartsbeyn M, Meehan SA. Piloleiomyomas in multiple cutaneous and uterine leiomyoma syndrome (hereditary leiomyomatosis and renal cell cancer or Reed syndrome). Dermatol Online J. 2015;21:13030/qt16s9k7bv.
72. Ristau BT, Kamat SN, Tarin TV. Abnormal Cystic Tumor in a Patient with Hereditary Leiomyomatosis and Renal Cell Cancer Syndrome: Evidence of a Precursor Lesion? Case Rep Urol. 2015;2015:303872.
73. Ritzmann S, Hanneken S, Neumann NJ, Ruzicka T, Kruse R. Type 2 segmental manifestation of cutaneous leiomyomatosis in four unrelated women with additional uterine leiomyomas (Reed's Syndrome). Dermatology. 2006;212:84-7.
74. Romero-Pérez D, Encabo-Durán B, Blanes-Martínez M, Niverio-de Jaime M. Papules on the arms and renal cell carcinoma. Clin Exp Dermatol. 2017;42:806-8.
75. Rongioletti F, Fausti V, Ferrando B, Parodi A, Mandich P, Pasini B. A novel missense mutation in fumarate hydratase in an Italian patient with a diffuse variant of cutaneous leiomyomatosis (Reed's syndrome). Dermatology. 2010;221:378-80.
76. Russo T, Piccolo V, Staibano S, Alfano R, Mascolo M, Argenziano G. Image Gallery: Segmental cutaneous leiomyomas in a patient with Reed syndrome. Br J Dermatol. 2016;175:e123.
77. Serra D, Amaro P, Gonçalo M, Silva M, Ferrando B, Pasini B, et al. Gastric leiomyoma and hyperplastic polyposis coli in a patient with multiple cutaneous and uterine leiomyomatosis. J Cutan Med Surg. 2012;16:208-11.
78. Smith S, Casady M, Driscoll MS. Novel mutation in the fumarate hydratase gene in a patient with Reed syndrome. Dermatol Online J. 2015;21:13030/qt56h2h20t.
79. Smith SC, Sirohi D, Ohe C, McHugh JB, Hornick JL, Kalariya J, et al. A distinctive, low-grade oncocytic fumarate hydratase-deficient renal cell carcinoma, morphologically reminiscent of succinate dehydrogenase-deficient renal cell carcinoma. Histopathology. 2017;71:42-52.
80. Sommer LL, Schnur RE, Heymann WR. Melanoma and basal cell carcinoma in the hereditary leiomyomatosis and renal cell cancer syndrome. An expansion of the oncologic spectrum. J Dermatol Case Rep. 2016;10:53-5.
81. Srivastava KP, Bajaj AK. Reed's Syndrome. Indian J Dermatol. 2012;57:156-7.
82. Tan RYP, Walsh M, Howard A, Winship I. Multiple cutaneous leiomyomas leading to discovery of novel splice mutation in the fumarate hydratase gene associated with HLRCC. Australas J Dermatol. 2017;58:e246-8.
83. Teh J, Kinnear N, Douglass-Molloy H, Hennessey DB. Hereditary leiomyomatosis and renal cell cancer syndrome: a family affair. BMJ Case Rep. 2017;2017:bcr2016218270.
84. Toro JR, Nickerson ML, Wei MH, Warren MB, Glenn GM, Turner ML, et al. Mutations in the fumarate hydratase gene cause hereditary leiomyomatosis and renal cell cancer in families in North America. Am J Hum Genet. 2003;73:95-106.
85. Trpkov K, Hes O, Agaimy A, Bonert M, Martinek P, Magi-Galluzzi C, et al. Fumarate Hydratase-deficient Renal Cell Carcinoma Is Strongly Correlated With Fumarate Hydratase Mutation and Hereditary Leiomyomatosis and Renal Cell Carcinoma Syndrome. Am J Surg Pathol. 2016;40:865-75.
86. Tsagoudis K, Magiera-Lappann I, Haverkamp T, Drebber U, Agaimy A, Tantcheva-Poόr I. Hereditary leiomyomatosis and renal cell cancer syndrome: A novel mutation in the FH gene. J Dermatol. 2018;45:373-5.
87. Tulandi T, Foulkes WD. Hereditary leiomyomatosis and renal cell cancer syndrome. CMAJ. 2016;188:140.
88. van der Pol CB, Lee FS, Fasih N. Case 221: Hereditary Leiomyomatosis and Renal Cell Cancer Syndrome. Radiology. 2015;276:922-7.
89. van Spaendonck-Zwarts KY, Badeloe S, Oosting SF, Hovenga S, Semmelink HJ, van Moorselaar RJ, et al. Hereditary leiomyomatosis and renal cell cancer presenting as metastatic kidney cancer at 18 years of age: implications for surveillance. Fam Cancer. 2012;11:123-9.
90. Varol A, Stapleton K, Roscioli T. The syndrome of hereditary leiomyomatosis and renal cell cancer (HLRCC): The clinical features of an individual with a fumarate hydratase gene mutation. Australas J Dermatol. 2006;47:274-6.
91. Vieregge EL, Kyei A. Multiple painful skin-colored papules. JAMA Dermatol. 2013;149:223.
92. Wang C, Tetzlaff M, Hick R, Duvic M. Reed syndrome presenting with leiomyosarcoma. JAAD Case Rep. 2015;1:150-2.
93. Wheeler KC, Warr DJ, Warsetsky SI, Barmat LI. Novel fumarate hydratase mutation in a family with atypical uterine leiomyomas and hereditary leiomyomatosis and renal cell cancer. Fertil Steril. 2016;105:144-8.
94. Wollina U, Schönlebe J. Reed's syndrome: segmental piloleimyomas type 1 and uterus myomatosus. J Dermatol Case Rep. 2014;8:67-9.
95. Woolner K, O'Toole A, LaBerge L. First Presentation of Hereditary Leiomyomatosis and Renal Cell Cancer Syndrome in Pregnancy. J Cutan Med Surg. 2016;20:334-6.
96. Yaldiz M, Metin M, Erdem MT, Dikicier BS, Kahyaoglu Z. Two sisters with Reed's syndrome: treatment with pregabalin. Dermatol Online J. 2015;21:13030/qt4wx2s2xm.
97. Young KZ, Raisanen TD, Else T, Harms PW, Cha KB. A few pink papules in an adult woman: Incidental finding leads to diagnosis of hereditary leiomyomatosis and renal cell cancer. JAAD Case Rep. 2019;5:419-21.
